# A Case of Diffuse Traumatic Cellulitis*Read before the Buffalo Medical Union.

**Published:** 1883-05

**Authors:** B. H. Daggett

**Affiliations:** Buffalo, N. Y.


### Clinical Reports.

### A Case of Diffuse Traumatic Cellulitis.*

By B. H. Daggett, M. D., Buffalo, N. Y.

Laundry, a ship-carpenter, thirty-eight years old, temperate habits, health impaired by a chronic disease, struck himself upon the back of the hand over the second metacarpal bone. He reported to me the second day after his injury for examination, and I found a contusion at the seat of the injury without a break of the skin. Eight days afterwards I was invited to take charge of the case, and I found him suffering severely and greatly prostrated. The hand and forearm were extremely swollen, the skin was cold, mottled and boggy, the hand crepitated under pressure, vesication had denuded its dorsal surface, and sphacelation was exposing the second and third metacarpal bones and extending down the corresponding fingers.

The periosteum was intact. An ichorous discharge with shreds of sloughy, necrosed tissue was bathing the parts. Deep-seated suppuration could be detected in the palm of the hand and thick pus was oozing from a small cut opposite the base of the first

* Read before the Buffalo Medical Union.

finger. Putting my probe into this cut it passed freely across the hand beneath the deep palmar facia. The fingers were so distorted by œdematus swelling, that a bystander asked if they were not broken.

The patient was etherized, and incisions deep and free were made down the back of the hand over the second and third metacarpal bones, and down the corresponding fingers to the middle joints. The palm was also operned by two incisions. From these cuts there flowed nearly a quart of inflammatory blood, serum and pus. The hand was bathed in carbolized water and the same was injected into the excavated palm, and both hand and arm were enveloped in a generous antiseptic poultice, which was to be renewed every four hours. An anodyne when required, quinine, ferric chloride and alcoholic stimulants with a suitable diet were prescribed.

From this time improvement was gradual but continuous, and in nine weeks the hand had healed except between the fingers. The third and fourth fingers would close nearly into the palm of the hand, and the first and second fingers would readily brush off the end of the thumb.

In three months, Laundry was again at work at his trade. Prompt and radical measures had checked in its course a deep-seated, rapidly-spreading, non-self-limiting, inflammatory affection which had induced great systemic depression, local sphacelation, and was threatening not only the integrity of the limb, but life itself. In this connection, and for the purpose of contrasting the course of a phlegmonous erysipelas or cutaneo-cellulitis, I will relate the following brief history:

Mr. T., forty-eight years of age, full habit, florid skin, a free liver, temperate habits, robust health, sprained his ankle in California, and continued on his journey home, not giving it needed rest. I saw him on his return, and observed that he if kept quiet and the limb elevated, the swelling about the ankle and the erythymatous blush which extended to the knee would subside, and that dependence of the limb with slight use would restore the redness and swelling. He was put to bed one night with his limb in a hydropathic pack, and got up in the morning saying, "I am cured in a night." Thirty minutes' use of the limb renewed the swelling and redness. This manner of using the limb was continued in spite of protests, until the inflammatory action extended to the cellular tissue, resulted in suppuration, thus producing a cutaneo-cellulitis or a phlegmonous erysipelas. The exciting cause in both cases was traumatism without septic or wound-poisoned infection. They were allied in character, but differed in the degree of their severity and in their course. In the first case, the cellular tissue was primarily affected, the skin suffering later by being cut off from its base of supplies. In the second, the skin was primarily affected, the inflammation extending therefrom to the cellular structure. In the following case the exciting cause of the cellulitis is evident:

J. C., a laborer, thirty years old, doubtful habits, good health, crushed the middle finger of the right hand at first joint, beneath the flange of a barrel of oil, and refused to have it amputated. Four days afterwards he called upon me, and I found the finger below the injury gangrenous, and above swollen and inflamed, and I amputated at the second joint. The next day the stump and hand were greatly swollen. The flaps were freed and the hand bathed in carbolized water and poulticed. One of the flaps sloughed and the disease was arrested only after free incisions. In cases one and two, was there some peculiar condition of the system which received from without or begot within itself the materies morbi?

Laundry was debilitated by a chronic malady, and he lived near the historic Hamburgh canal, without even a friendly fence intervening. The ground is low, wet and unsewered, and kitchen slops, accumulations of decaying animal and vegetable matter, with surface drainage, flow lazily in open gutters. A favorable subject and a fruitful field for the propagation of disease-breeding germs! The etymology of the word erysipelas indicates that it is derived from words which mean reddening of the skin or a spreading redness, so that an attribute of two forms of erysipelous diseases gives the name to this important class of maladies. The nosonomy of these affections could be properly revised. The word or name cutitis, for skin erysipelas; cutaneo-cellulitis, for phlegmonous erysipelas, and cellulitis would differentiate as between themselves and designate the structures affected and their inflamed condition. Until the special poison or condition of the system which begets these maladies is known and demonstrated, we can only name and treat conditions. Cellulitis has been fully described by Lawrence, Duncan, Nunelly, and their views have been endorsed by later authorities.

It is a graver disease than its fellows — skin and phlegmonous erysipelas — and is characterized by more prostration and nervous excitement. Its onset may be insidious or violent. Its course is not *self*-limiting. "Often the skin gives no clue to the condition beneath." Traumatic cellulitis spreads by continuity of tissue from the seat of injury; septic or wound-poisoned cellulitis attacks remote parts, between which and the puncture no direct connection can be traced. Metastasis is a concomitant of septic or wound-poisoned cellulitis. In relation to the local treatment, the most important indication is to make early and free incisions to permit the escape of the irritating inflammatory products, and which also induces local depletion.

